# Assessment of Clopidogrel on the Left Ventricular Ejection Fraction in Acute Myocardial Infarction

**Published:** 2010

**Authors:** Pejman Khosravi, Hasan Shemirani, Behnoud Hedayatpour, Allahyar Golabchi

**Affiliations:** 1 Clinical Resident, Department of Internal Medicine, School of Medicine, Isfahan University of Medical Sciences (IUMS), Isfahan, Iran; 2 Associate Professor, Department of Cardiology, School of Medicine, IUMS, Isfahan, Iran; 3 Medicine Doctor, Isfahan, Iran; 4 Clinical Resident, Department of Cardiology, School of Medicine, IUMS, Isfahan, Iran

**Keywords:** Heart failure, Myocardial infarction, Prevention

## Abstract

**Objectives:**

Left ventricular (LV) dysfunction heart failure is one of the causes of morbidity and mortality following ST elevation myocardial infarction (STEMI). This study was done to determine the clopidogrel effect in preventing reduced LV function in patients with STEMI.

**Methods:**

In this study, 144 patients with STEMI admitted to the Isfahan University of Medical Sciences hospitals were followed in two groups for one month. The case group received Clopidogrel, 300 mg, on admission and then, 75 mg daily, while the control group received routine therapy for STEMI without Clopidogrel. Left ventricular ejection fraction (LVEF) on the 4^th^ day and one month after STEMI was measured by echocardiography. The results of LVEF were compared within and between groups.

**Results:**

The mean LVEF in the case group on the 4^th^ day and one month after STEMI were 45.92 and 52.15%, respectively (P<0.001). The mean LVEF in the control group on 4^th^ day and one month after STEMI were 44.72 and 42.71%, respectively.

**Conclusions:**

We suggest that Clopidogrel is effective in prevention of LVEF reduction in patients with STEMI.

## INTRODUCTION

Antiplatelet therapy has been shown to significantly reduce the risk of serious vascular events in high-risk patients, including those with a prior acute ischemic event.^1^ The long-term use of antiplatelet agents is thus a key component of secondary prevention measure following acute coronary syndromes (ACS). Platelets play a fundamental role in the pathophysiology of unstable angina and acute myocardial infarction (MI) as plaque rupture is followed by adhesion, activation and aggregation of platelets, and formation of a coronary thrombus.^2^ Platelet activation also plays a role in the development of secondary ischemia following an initial ACS event and is more pronounced following intracoronary stent implantation than after coronary balloon angioplasty alone.^3^ Secondary ischemia, which may include MI and stroke, is a significant hazard for patients who have already experienced an acute event.^4^,^5^ Some of the strongest evidence available for long-term prevention of adverse cardiac events in patients with coronary disease pertains to the use of aspirin.^1^,^6^ Current treatment guidelines from the American College of Chest Physicians (ACCP) and jointly issued guidelines from the American Heart Association (AHA) and American College of Cardiology (ACC) recommend that aspirin therapy be continued indefinitely for secondary prevention of ischemic events following ACS, with the Clopidogrel to be added for up to 12 months for the majority of patients with unstable angina, non-ST segment elevation MI (NSTEMI), or ST segment elevation MI (STEMI),^7^–^10^ or longer in the case of patients receiving drug-eluting stents.^9^,^11^

## METHODS

This controlled clinical trial was conducted in 3 hospitals of Isfahan University of Medical Sciences from April 2008 to October 2009. Patients were eligible if they had typical chest discomfort for more than 20 minutes, ST-segment elevation of at least 0.1 mV in at least two contiguous limb leads or ST-segment elevation of at least 0.2 mV in at least two contiguous precordial leads. We excluded patients with cardiogenic shock, major surgery within the previous 6 months, ongoing bleeding or bleeding diathesis, previous stroke in the last 6 months, emergency requirement to PCI and patients who had bleeding after infusion of streptokinase. As per protocol, all patients were pretreated with aspirin (325 mg orally as bolus and 100 mg daily) and strep-tokinase. Surface ECGs with 12 leads were recorded before and 90 min after streptokinase infusion for ST-segment resolution (STR) and if patients required emergency PCI, they were excluded.

### 

#### Intervention

Group I included 72 patients that were treated by usual treatment and Clopidogrel, administered 300 mg orally as bolus on the first day, and 75 mg orally, daily for at least 1 month. Group II involved those who were treated before 2008 with standard protocol and not received Clopidogrel (n=72)^17^. After streptokinase infusion, we evaluated the patients by echocardiography after 4 days and 1 month.

The assessed echocardiography end point included left ventricular ejection fraction (LVEF). It was performed through Biplane Simpson technique by one specialist with similar set and situation. In this study, the mean LVEF after 4 days and 1 month were determined in both groups and were compared.

#### Statistical Analysis

Statistical analysis was performed with the SPSS 16 program. All continues variable were reported as mean ± SD. Categorical variables were compared using Mann-Whitney U test. The differences of quantitative variables between the two groups were compared using t-tests. A value of P<0.05 was considered significant.

## RESULTS

A total of 153 patients were screened and 144 patients were enrolled in the study. Two patients with cardiogenic shock, 4 patients with emergency PCI requirement, 1 patient with history of stroke in the last 6 months and 2 patients with history of major surgery within the previous 6 months were excluded. There were no significant differences in baseline characteristics such as age, sex, current smoking status between the two groups but the number of patients with diabetes mellitus in group I were significantly higher than that in group II (P=0.01, Table 1).

**Table 1:** Baseline characteristics of patients.

Characteristics	Group I (N=72)	Group II (N=72)	P
Age (year)	58.22 ± 9.6	57.58 ± 13.42	0.7
Sex (male) (%)	42 (58.3%)	51 (70.8%)	0.1
Current smoker (%)	33 (45.80%)	37 (51.3%)	0.3
Diabetes mellitus (%)	34 (47.2%)	20 (27.7%)	0.01

Group I: Clopidogrel group.

Group II: non-Clopidogrel group.

The mean LVEF after 4 days and 1 month were determined in both groups. In Clopidogrel group, the mean LVEF after 1 month significantly increased compared with that after 4 days (P=0.001). In non-Clopidogrel group, the mean LVEF after 1 month non-significantly decreased compared with the mean LVEF after 4 days (P=0.05, Table 2).

**Table 2:** Comparison of LVEF after 4 days and one month in the two groups studied

	LVEF after 4 days (%)	LVEF after 1 month (%)	P
Group I	45.92 ± 10.36	52.15 ± 9.85	0.001
Group II	44.72 ± 9.95	42.71 ± 12.38	0.05

LVEF= left ventricular ejection fraction

In Clopidogrel group, the results of LVEF after 4 days and 1 month in diabetic and non-diabetic patients, current smokers and non-smoker are given in Table 3. The mean LVEF in diabetic patients increased 5.27% and in non-diabetic patients increased 7.11%, Therefore, diabetes mellitus is the cause of retardation of remission of heart function after acute MI. The increment of LVEF in non-smokers was more than that in current smokers. Therefore, the smoking is the cause of coronary vessels occlusion and has negative effects on heart function.

**Table 3:** Comparison of LVEF by diabetes and smoking status in Clopidogrel group

Clopidogrel Group	LVEF after 1 month (%)	LVEF after 4 days (%)	P
Diabetic patients	50.15 ± 11.57	44.88 ± 10.82	0.001
Non-diabetic patients	53.95 ± 7.72	46.84 ± 11.57	0.003
Current smokers	48.64 ± 10.02	43 ± 10.92	0.004
Non-smokers	± 8.77	48.38 ± 9.31	0.001

LVEF= left ventricular ejection fraction

## DISCUSSION

The antiplatelet agents are effective in reducing the risk of a second ischemic event in patients with atherothrombotic disease. The Clopidogrel is now recommended as dual therapy with aspirin in secondary prevention for patients with both ST segment and non-ST segment ACS, including those undergoing PCI.^1^ Clopidogrel is a thienopyridine with a faster onset of action and more potent antiplatelet effect than older agents. It has shown superiority over the currently accepted standard in reducing the risk of the composite end point of cardiovascular death, nonfatal MI, and nonfatal stroke in patients who undergo PCI following ACS at standard dosage, as well as reducing the risk of the component end points of nonfatal MI, urgent targetvessel revascularization, and stent thrombosis.^5^ The greatest benefit appears to be in patients with diabetes mellitus. However, the enhanced antiplatelet activity and greater efficacy seen with Clopidogrel in clinical trials has been accompanied by increased bleeding risk. The FDA advisory committee voted unanimously for the approval of Clopidogrel along with providing enough guidance to physicians about the increased risk of this medication in low-weight or elderly patients and avoidance of its use around CABG, other surgical or invasive procedures and in patients with prior or current stroke or TIA.^6^,^7^ Identification of patients at higher risk of bleeding events and attention to discontinuation of therapy before surgery may help guide therapeutic decisions and optimize outcomes including the benefitrisk ratio.

## References

[1] (2002). Collaborative meta-analysis of randomised trials of antiplatelet therapy for prevention of death, myocardial infarction, and stroke in high risk patients. BMJ.

[2] Yeghiazarians Y, Braunstein JB, Askari A, Stone PH (2000). Unstable angina pectoris. N Engl J Med.

[3] Inoue T, Sohma R, Miyazaki T, Iwasaki Y, Yaguchi I, Morooka S (2000). Comparison of activation process of platelets and neutrophils after coronary stent implantation versus balloon angioplasty for stable angina pectoris. Am J Cardiol.

[4] Steg PG, Bhatt DL, Wilson PW et al (2007). One-year cardiovascular event rates in outpatients with atherothrombosis. JAMA.

[5] Yusuf S, Zhao F, Mehta SR, Chrolavicius S, Tognoni G, Fox KK (2001). Effects of Clopidogrel in addition to aspirin in patients with acute coronary syndromes without ST-segment elevation. N Engl J Med.

[6] Anderson JL, Adams CD, Antman EM et al (2007). ACC/AHA 2007 guidelines for the management of patients with unstable angina/non ST-elevation myocardial infarction: a report of the American College of Cardiology/American Heart Association Task Force on Practice Guidelines (Writing Committee to Revise the 2002 Guidelines for the Management of Patients With Unstable Angina/Non ST-Elevation Myocardial Infarction): developed in collaboration with the American College of Emergency Physicians, the Society for Cardiovascular Angiography and Interventions, and the Society of Thoracic Surgeons: endorsed by the American Association of Cardiovascular and Pulmonary Rehabilitation and the Society for Academic Emergency Medicine. Circulation.

[7] (2005). 2005 American Heart Association Guidelines for Cardiopulmonary Resuscitation and Emergency Cardiovascular Care. Circulation.

[8] Antman EM, Hand M, Armstrong PW (2008). Focused Update of the ACC/AHA 2004 Guidelines for the Management of Patients With ST-Elevation Myocardial Infarction: a report of the American College of Cardiology/American Heart Association Task Force on Practice Guidelines: developed in collaboration With the Canadian Cardiovascular Society endorsed by the American Academy of Family Physicians: 2007 Writing Group to Review New Evidence and Update the ACC/AHA 2004 Guidelines for the Management of Patients With ST-Elevation Myocardial Infarction, Writing on Behalf of the 2004 Writing Committee. Circulation.

[9] Becker RC, Meade TW, Berger PB et al (2008). The primary and secondary prevention of coronary artery disease: American College of Chest Physicians Evidence-Based Clinical Practice Guidelines. Chest.

[10] Hirsh J, Guyatt G, Albers GW, Harrington R, Schunemann HJ (2008). Executive summary: American College of Chest Physicians Evidence-Based Clinical Practice Guidelines (8th Edition). Chest.

[11] King SB, Smith SC, Hirshfeld JW (2008). 2007 Focused Update of the ACC/AHA/SCAI 2005 Guideline Update for Percutaneous Coronary Intervention: a report of the American College of Cardiology/ American Heart Association Task Force on Practice Guidelines: 2007 Writing Group to Review New Evidence and Update the ACC/AHA/SCAI 2005 Guideline Update for Percutaneous Coronary Intervention, Writing on Behalf of the 2005 Writing Committee. Circulation.

